# Hydrogel Matrix Containing Microcarriers for Dexamethasone Delivery to Protect Against Cisplatin-Induced Hearing Loss

**DOI:** 10.7759/cureus.71142

**Published:** 2024-10-09

**Authors:** Maximilian G Dindelegan, Cristina M Blebea, Maria Perde-Schrepler, Violeta Necula, Alma A Maniu, Violeta Pascalau, Catalin Popa, Sergiu Susman, Luciana M Gherman, Anca D Buzoianu

**Affiliations:** 1 Department of Clinical Pharmacology, “Iuliu Hațieganu” University of Medicine and Pharmacy, Cluj-Napoca, ROU; 2 Department of Surgery – Practical Abilities, “Iuliu Hațieganu” University of Medicine and Pharmacy, Cluj-Napoca, ROU; 3 Department of Otolaryngology, Head and Neck Surgery, Institute of Oncology “Prof. Dr. Ion Chiricuță”, Cluj-Napoca, ROU; 4 Department of Otorhinolaringology, "Iuliu Hațieganu” University of Medicine and Pharmacy, Cluj-Napoca, ROU; 5 Department of Otorhinolaringology, “Iuliu Hațieganu” University of Medicine and Pharmacy, Cluj-Napoca, ROU; 6 Department of Radiobiology and Tumoral Biology, Institute of Oncology “Prof. Dr. Ion Chiricuță”, Cluj-Napoca, ROU; 7 Department of Materials Science and Engineering, Technical University of Cluj-Napoca, Cluj-Napoca, ROU; 8 Department of Morphological Sciences, “Iuliu Hațieganu” University of Medicine and Pharmacy, Cluj-Napoca, ROU; 9 Department of Pathology, IMOGEN Research Center, Cluj-Napoca, ROU; 10 Laboratory Animal Facility – Centre for Experimental Medicine, “Iuliu Hațieganu” University of Medicine and Pharmacy, Cluj-Napoca, ROU; 11 Department of Clinical Pharmacology, "Iuliu Hațieganu" University of Medicine and Pharmacy, Cluj-Napoca, ROU

**Keywords:** cisplatin, dexamethasone, hearing loss, hydrogel, microparticles, ototoxicity, rat

## Abstract

A functional hydrogel containing biopolymer microcarriers loaded with dexamethasone was developed to address the hearing loss that results from cisplatin ototoxicity. The drug delivery platform was tested both in vitro in the HEI-OC1 inner ear cell line and in vivo in a rat animal model. The newly described formula offered prolonged release of the contained dexamethasone for up to six days and transformed into a solid state at body temperature, thus counteracting its clearing through the Eustachian tube when injected into the middle ear. When tested in vitro, the inner ear cells exposed to cisplatin showed significantly higher viability at 48 hours when seeded on hydrogel containing dexamethasone-loaded microparticles than the cells treated with free dexamethasone. In the rat in vivo model, the ears of the rats treated with the hydrogel formulation presented better hearing thresholds after cisplatin administration than contralateral ears treated with free dexamethasone. The ears of the rats treated with microcarriers without inclusion in the functional hydrogel obtained better results than the dexamethasone treatment group but not as good as the hydrogel-containing microcarrier group. Histological assessment of the rats’ inner ears showed better integrity of the structures and lower apoptosis in the microcarrier-treated groups than in the control group. Overall, the newly described microcarrier of dexamethasone offers better protection against cisplatin-induced hearing loss than free dexamethasone, especially when contained in a functional hydrogel formulation.

## Introduction

Hearing loss is a common chronic condition affecting all age groups, with a higher prevalence in the older population. Over 60% of people suffering from hearing impairment are older than 50 years of age [1]. The consequences of hearing loss range from intellectual deterioration to depression, anxiety, and even a higher risk of developing dementia [2]. Sensorineural hearing loss (SNHL) is caused by impairment of the cochlea through cell destruction in the inner ear or destruction of the retrocochlear pathways, which transmit neural signals to the brain [3]. It can be inherited or acquired. The onset of acquired SNHL can be rapid, such as in the case of sudden sensorineural hearing loss, where the decline in hearing happens over several hours up to a few days; of medium duration, such as a few weeks in the case of hearing loss induced by ototoxic drugs (e.g., platinum-based chemotherapeutic drugs); or even acquired over multiple years, for example, through exposure to excessively loud sounds leading to noise-induced hearing loss [4].

Ototoxicity refers to hearing loss resulting from inner ear sensorineural cell damage after therapy with different drugs such as diuretics, aminoglycosides, platinum-based chemotherapeutic agents, non-steroidal anti-inflammatory drugs, and others. The drugs with the highest ototoxic potential are represented by aminoglycosides and antineoplastic drugs, especially cisplatin [5]. The common mechanism of different molecules that cause ototoxicity is the formation of high levels of reactive oxygen species [6]. Hearing loss usually starts in the highest audible frequencies, damaging the cells located in the basal turn of the cochlea where high frequencies are processed [7].

Platinum-based chemotherapy is used to treat a multitude of malignancies, either as monotherapy or in combination with other therapeutic alternatives. Cisplatin is considered a very effective, relatively cheap, and accessible drug, so it is commonly used in malignancy treatment plans for head and neck squamous cell carcinoma, lung cancer, lymphoma, ovarian cancer, testicular cancer, and other types of cancers [8]. It is also more ototoxic than carboplatin, with its ototoxicity having been identified during the 1980s [5]. It damages the structures of the inner ear, with hair cell destruction in the organ of Corti being the most characteristic damage [9]. Cisplatin-associated toxicity commonly presents as progressive, permanent, bilateral, and sensorineural hearing loss, mostly affecting the higher frequencies and associated with tinnitus [10]. The toxicity is proportional to the number of treatment sessions, duration, and total cumulative dose, with age also playing an important role [11]. Approximately 60% of oncological patients undergoing chemotherapy with cisplatin suffer from permanent hearing loss [12]. Experimental studies conducted in rodents have shown that dexamethasone (Dexa) injection protects against cisplatin-induced ototoxicity in experimental settings [13].

The administration routes available to physicians in clinical settings are represented by systemic and local (intratympanic) routes. Each of these routes has its own advantages and disadvantages. The systemic route represents a less complex modality of drug administration. This administration method is not as uncomfortable and poses no risk of persistent tympanic membrane perforation or middle ear infection. On the other hand, the intratympanic administration of drugs, even if not as comfortable to the patients, poses the risks mentioned before and the administration must be repeated multiple times during the treatment duration due to the clearance of the drugs from the middle ear through the Eustachian tube and via resorption [14]. However, locally administrating drugs presents clear advantages over systemic administration. The advantages of intratympanic delivery reflect the fact that the blood-labyrinth barrier of the inner ear is bypassed, and the substance can reach higher concentrations through diffusion directly across the round and oval window membranes into the inner ear [3]. Due to the nature of local delivery, the drugs will not cause systemic side effects and some of their contraindications are not applicable [15]. Different prolonged and targeted drug delivery systems have been studied for intratympanic delivery of corticotherapeutic agents [16,17].

Our research team has previously studied the preparation and use of a biopolymer microcarrier loaded with dexamethasone, which was capable of offering prolonged release of dexamethasone for up to six days [18]. In the current experimental study, we aimed to test the previously described microcarrier and a new iteration of it, namely, a functional hydrogel containing the Dexa-loaded microparticles, both in vitro and in vivo. For the in vitro experiment, we used the HEI-OC1 inner ear cell line, and for the in vivo experiments, we evaluated whether the microcarriers offer better auditory brainstem response (ABR) thresholds and better histological findings than Dexa solution in an experimental rat model of cisplatin-induced ototoxicity.

## Materials and methods

Chemicals

Phosphatidylcholine from Soybean Lipoid S 100 was kindly provided by Lipoid GmbH (Ludwigshafen, Germany). Dexamethasone powder, dimethyldioctadecildiammonium bromide (≥98%), bovine serum albumin (BSA), albumin fraction V for biochemistry, calcium chloride, pectin from citrus peel (≥74%), and galacturonic acid were all purchased from Merck (Darmstadt, Germany). VitroGel was obtained from TheWell Bioscience Inc., North Brunswick, NJ, USA. Sterile-filtered and endotoxin-tested Dulbecco’s phosphate-buffered saline (PBS) and all reagents used in biological studies, including Dulbecco’s modified Eagle medium (DMEM) and fetal bovine serum (FBS), were purchased from Sigma Life Science/Merck (Darmstadt, Germany). Alamar Blue was purchased from Thermo Fischer Scientific (Waltham, MA USA). Ethanol (96%) was purchased from S.C. Nordic Chemicals SRL (Cluj-Napoca, Romania). Dexamethasone phosphate solution (4 mg/ml) was purchased from Rompharm Company SRL (Otopeni, Romania). Cisplatin aqueous infusion solution was purchased from Actavis (Teva Pharmaceutical Industries, București, Romania; CDDP, 1 mg/ml).

Hematoxylin and eosin (H&E) reagents were purchased from Heinz Herenz (Hamburg, Germany). Anti-caspase-3 antibody (EPR18297) ab184787, clone number EPR18297, was purchased from ABCAM (Cambridge, UK) and the reagents used for the immunohistochemistry were provided by Master Diagnostica (Spain).

Preparation of the hydrogel containing Dexa-loaded lipid/pectin/BSA microparticles (Vitrogel-Lmp/Dexa)

The Dexa-loaded microparticles, Lmp/Dexa, were prepared as described in our previous work [18]. Briefly, a 0.2% pectin solution, obtained via dissolution in distilled water under vigorous magnetic stirring (1000 rpm) with heating up to 90 °C, filtration on yellow filter paper, and cooling at room temperature, was mixed with 0.1% BSA water solution in the ratio of 2:1 (w/w) until it homogenized. The mixture was incubated at 30 °C under slow magnetic stirring (200 rpm). A 1% CaCl2 (10% CaCl2 relative to the solid pectin) aqueous solution was added under continuous stirring. A multi-component ethanolic solution was prepared by successively adding various components, after the previous one had completely dissolved, in the following order: Dexa, Lipoid S100, DDAB in Ethanol 96%. Under agitation at room temperature, Dexa:Lipoid S100:DDAB in the ratio of 2:10:1, 3:10:1, or 4:10:1 (w/w) was added drop-wise using a 2 ml syringe and then incubated for 15 min without stirring after complete addition. The suspension containing Lmp/Dexa was kept at 4 °C for a minimum of 24 hours for deposition. After removal of the supernatant, for each Lmp/Dexa formulation, the hydrogel based on Vitrogel was prepared by mixing Lmp/Dexa suspension with Vitrogel in the 1:1 (vol/vol) ratio. 

In vitro release study of Vitrogel-Lmp/Dexa

The study was performed under the same conditions as the Dexa release study using Lmp/Dexa in our previously published research [18]. The release behavior of the hydrogel matrix compared with that of Lmp/Dexa has been studied, as well as the influence of the Dexa concentration in the hydrogel on the release profile. The in vitro release of Dexa from Vitrogel-Lmp/Dexa was conducted in PBS pH 7.4 containing 1% ethanol to allow for solubilization of Dexa. The assessment of Dexa concentration in the release medium using UV-Vis Quant mode based on the calibration curve was performed. The Dexa release profile from the Lmp/Dexa hydrogel was obtained using a previously reported dialysis method [19] at 37 °C, under slow magnetic stirring at 150 rpm. Briefly, 0.6 ml of Vitrogel-Lmp/Dexa sample preheated at 37 °C was introduced into a dialysis bag (MWCO:3 kDa), and the sealed bag was immersed in 20 ml 1% ethanol solution (i.e., the release medium) and then placed into a 100 ml Erlenmeyer flask with a flat bottom and glass ground stopper for 144 hours. At certain time intervals, three samples (each 1 ml) of release medium were removed and replaced with the same volume of fresh medium. The sample was further incubated. The collected samples were diluted with 50% ethanol solution (v/v), homogenized, and then introduced in the spectrometer measuring cuvette for Dexa quantification at λ = 243 nm against the adequate previously registered calibration curve. All studies were made in triplicate. The Dexa concentrations at any specified time tn _(1→32)_ were determined (t_1_ = 5 min, t_2_ = 10 min, t_3_ = 15 min, t_4_ = 30 min, t_5_ = 45 min, t_6_ = 1 hr, t_7 _= 2 hr, t_8_ = 3 hr, t_9_ = 4 hr, t_10_ = 5 hr, t_11 _= 6 hr, t_12_ = 12 hr, t_13_ = 24 hr, t_14_ = 30 hr, t_15 _= 36 hr, t_16 _= 42 hr, t_17_ = 48 hr, t_18_ = 54 hr, t_19 _= 60 hr, t_20 _= 66 hr, t_21 _= 72 hr, t_22_ = 78 hr, t_23_ = 84 hr, t_24_ = 90 hr, t_25 _= 102 hr, t_26_ = 108 hr, t_27_ = 114 hr, t_28_ = 120 hr, t_29 _= 126 hr, t_30 _= 132 hr, t_31 _= 138 hr, and t_32_ = 144 hr) (C_n_).

The cumulative Dexa percentage was calculated as the percentage of Dexa released at a specific time $n$ against the amount of Dexa estimated in the sample subjected to release, based on Dexa Loadingef (%) (Equation 1):

\[ \text{Cum Dexa rel (%)} = \frac{\text{Quantum of Dexa at specific time } (t_n) \times 100}{\text{Total feeding Dexa}} \tag{1}\]

The Quantum of Dexa at a specific time $t_n$, denoted as $m_n$, was calculated using Equation (2):

\[ m_n = C_n V_0 \tag{2} \]

The cumulative Dexa released quantum ($m_n$ cumulative) was calculated with Equation (3):

\[ m_n \text{ cumulative} = V \left( C_1 + C_2 + \ldots + C_{n-1} \right) + V_0 C_n \tag{3} \]

where V_0_ is the total volume of the release medium and V is the removed and replaced volume. In small volumes of the release medium, it was necessary to apply some corrections on the determined Dexa concentration in order to calculate the cumulative released Dexa, using Equation (3). It is necessary to take into account the Dexa quantity in the previously removed volume for analysis, which has to be added to the released Dexa calculation.

The Total Dexa before release was calculated by Equation (4):

\[ \text{Total Dexa before releasing} = \frac{\text{Dexa loadingef (%)} \times m}{100} \tag{4} \]

The cumulative release (%) of Dexa from the total Dexa before release was further plotted against the release time.

In vitro experiment on the HEI-OC1 cellular line

For the in vitro experiments, we used the HEI-OC1 cell line, isolated from the organ of Corti of transgenic “Immortomouse,” which was cultured under the following permissive conditions: 33°C, 10% CO_2_, in high-glucose DMEM supplemented with 10% FBS (all from Sigma-Aldrich/Merck KGaA, Darmstadt, Germany). 

Alamar Blue viability test

Vitrogel is a xeno-free (with no animal origin components) hydrogel system modified with a cell adhesive peptide RGD to promote cell attachment and cell-matrix interactions. Vitrogel solution is stable at room temperature, free-flowing, and transforms into a hydrogel matrix under physiological conditions. Vitrogel-Lmp/Dexa was prepared as described earlier.

Six wells of a 96-well plate (Nunc, Thermo-Fisher, Waltham, MA USA) were treated with 10 µl Vitrogel-Lmp/Dexa with a dexamethasone concentration of 15 µg (Vitrogel-Lmp/Dexa 15), while another six wells were treated with Vitrogel-Lmp/Dexa 15 diluted 1:2 with culture medium, thus containing 7.5 µg dexamethasone (Vitrogel-Lmp/Dexa 7.5). The plates were left overnight in the incubator at 37°C in order to solidify.

On the next day, HEI-OC1 cells were seeded at 2 × 10^4^ cells in 100 µl medium/well into the Vitrogel-Lmp/Dexa treated wells, as well as wells without Vitrogel-Lmp/Dexa. After a 24-hour incubation period, the wells without Vitrogel were treated as follows: six wells were left as control, six wells with 15 µg dexamethasone (Dexa 15) in 100 µl medium, and six wells with 7.5 µg dexamethasone (Dexa 7.5) in 100 µl medium. After 30 minutes, in half (three) of the wells with each treatment, cisplatin was added (100 µM) and the cells were left to incubate for 24 hours. The concentrations were selected based on previous cytotoxicity tests [18]. In previous experiments conducted by our research team, we obtained an IC50 (reducing cell viability by 50%) of cisplatin on HEI-OC1 cells of 65.79 µM. We used a concentration of 100 µM cisplatin, which is well above the IC50 concentration, to induce important cell toxicity [20].

On the next day, 10 µl Alamar Blue solution was added to each well, and the emitted fluorescence was read after one hour on a BioTek Synergy 2 microplate reader. Alamar Blue contains resazurin, which, upon entering living cells, is reduced to resorufin, a red-colored and highly fluorescent compound (560 nm excitation/590 nm emission). To calculate the percentage of viable cells, the fluorescence emitted by the treated cells was divided by the fluorescence of control cells and then multiplied by 100. The experiment was repeated as described before, but with the fluorescence being read after a 48-hour incubation period.

Fluorescence microscopy

The HEI-OC1 cells were seeded into Lab-Tek Nunc 16-well chamber slides and treated in a similar way as in the Alamar Blue test. After 24 hours of incubation, the cells were washed twice with PBS and 100 µl 20 nM MitoRed solution (Sigma-Aldrich-Merck KGaA, Darmstadt, Germany) was added. MitoRed is a Rhodamine-based dye that is capable of penetrating the cell membrane and localizing in the mitochondria; based on its membrane potential, the dye emits red fluorescence. After incubation with MitoRed at 37°C for an hour, the cells were washed, fixed with 4% paraformaldehyde for 15 minutes, and washed again with PBS. The slides were examined using a Nikon 600 Eclipse fluorescence microscope equipped with a 560 nm filter and a Nikon DSQi2 monochrome camera. The images were analyzed using the NIS Elements imaging software (Nikon Corporation, Minato City, Tokyo, Japan). Viable cells showed red fluorescence in the cytoplasm.

In vivo experiment on Wistar rats

Fourteen male eight- to nine-week-old Wistar rats weighing between 250 and 300 grams were included in the in vivo randomized experimental study. All animals were in good general health and without any signs of ear pathology. Otoscopy using a 2.7 mm diameter rigid endoscope was performed, and only animals without any signs of external or middle ear problems were included in the study. The exclusion criteria were any signs of ear pathology that could be seen on the initial endoscopy. The study protocol was conducted according to European law regarding the welfare of experimental animals. All the animals were housed in our institution’s experimental facility in controlled and safe conditions, and the study protocol received the approval of the ethical committee from our institution and from the National Veterinary Health and Food Safety Authority (approval 316/30.05.2022). Each subject had both of their ears tested, so we could minimize the number of animals used, according to the 3R principle (replacement, reduction, and refinement) [21].

After selecting the animals that met our inclusion criteria, seven of each were randomly assigned to one of the two tested groups. Animals from both groups received an intramuscular injection in the right hind limb with a mixture of ketamine (80 mg/kg) and xylazine (8 mg/kg), according to our local anesthesia protocol. Then, 15 mg/kg cisplatin was administered intraperitoneally in each subject, together with 2 ml of NaCl 0.9% administered subcutaneously, in order to help prevent the renal toxicity caused by cisplatin.

ABR thresholds were determined in all the rats in both ears [22]. ABR thresholds were measured on the first day, prior to receiving any treatment, and on the seventh day following the cisplatin administration and intratympanic injection. 

The ABR measurements were conducted in a soundproof room using an Opti-Amp amplifier connected to the SmartEP system provided by Intelligent Hearing Systems (Miami, FL, USA). Both ears had their thresholds tested for pure tones of 32 kHz, 24 kHz, 16 kHz, 8 kHz, and click stimulus. The sounds were administered through earphones inserted into the external ear canal. The duration of the stimulus was 1.3 ms and the repetition rate was 20/s. The tone burst stimulus (for the specific frequencies) was tested using high-frequency transducers, while ER insert earphones were used for the click stimulus (both provided by Intelligent Hearing Systems). Subcutaneous stainless steel needle electrodes (Intelligent Hearing Systems) were inserted into each subject, which was positioned as follows: the negative (recording) electrode was placed in the retroauricular area, the positive (reference) electrode was placed on the vertex, and the last electrode, namely, the ground electrode, was placed on the left hind limb of the rats. Ipsilateral evoked potentials in each of the ears were obtained as an average of 1024 sweeps, progressively reducing the stimulus by 10 dB steps; furthermore, 5 dB steps were also included when ABR measurements showed a change in wave patterns. The threshold was considered the lowest stimulating level at which an ABR response could be identified and reproduced consistently.

In each group, the right ear was considered the studied ear, while the left ear was considered the control ear. In one of the groups, the rats received a 30 µL intratympanic injection of Lmp/Dexa (group 1), and in the other group, the rats received 30 µL intratympanic injection of Vitrogel-Lmp/Dexa (group 2). Both Lmp/Dexa and Vitrogel-Lmp/Dexa contained a 1.5 mg/ml dexamethasone concentration. In both groups, the Lmp/Dexa and Vitrogel-Lmp/Dexa solution were injected into the right ear. The left ears of the rats in both groups received a 30 µL intratympanic injection containing dexamethasone solution with the same 1.5 mg/ml concentration. The intratympanic injections were performed under direct visualization using the same 2.7 mm diameter endoscope, which was used to check the animals for ear pathology. Animals were kept in separate containers after administering the substances, with access to water and food. 

After the ABR assessment was undertaken at seven days, the rats were euthanized using ketamine/xylazine solution overdose, and their temporal bones were dissected and collected for histopathological examination. 

The ABR results were interpreted in a blinded manner by the same researcher who assessed the hearing threshold. The ABR threshold shift was calculated as the difference between the hearing thresholds on the seventh day of the study and the thresholds measured before any interventions. 

Histological examination

Hematoxylin and eosin stain (H&E) and immunohistochemistry were performed automatically on 3-μm-thick sections of formalin-fixed and paraffin-embedded tissues. For immunohistochemistry, we used MD Stainer (Master Diagnostica) using ethylenediaminetetraacetic acid (EDTA; pH = 9) for antigen retrieval. For the immunohistochemical assessment of procaspase-3 expression, the specimens were incubated overnight at 4 °C with anti-caspase-3 antibody (EPR18297) ab184787, clone number EPR18297 (ABCAM) at a 1:1000 dilution. Positive and negative controls were used, in line with the recommendation of the manufacturer. We assessed the percentage of procaspase-3 positive cells (0-100%) and immunostaining intensity (absent, poor, moderate, or intense). Images were analyzed with an Axiolab 5, Zeiss microscope and captured with an Axiocam 208 color camera system. The slides were read independently by two experienced pathologists with no knowledge of data regarding the animals used for experiments. In the case of divergent results, the slides were reviewed by both pathologists working together, until a consensus was reached. 

Statistics

 For the in vitro study in the HEI-OC1 cell line, we used GraphPad Prism 9 (GraphPad Software, San Diego, CA, USA). For comparisons between several groups, we applied two-way ANOVA, followed by Bonferroni multiple comparisons. For the in vivo study, ABR threshold shift statistical analysis was conducted using the same GraphPad Prism 9 software. For comparisons between the groups, we applied two-way ANOVA, followed by Bonferroni multiple comparisons test, and compared the differences in hearing threshold values obtained after and before cisplatin treatment administration in the left and right ear in each group. All of the data sets passed the Shapiro-Wilk test and showed a normal distribution of data prior to two-way ANOVA analysis. Differences were regarded as significant when p < 0.05.

## Results

In vitro release of Vitrogel-Lmp/Dexa

**Figure 1:**
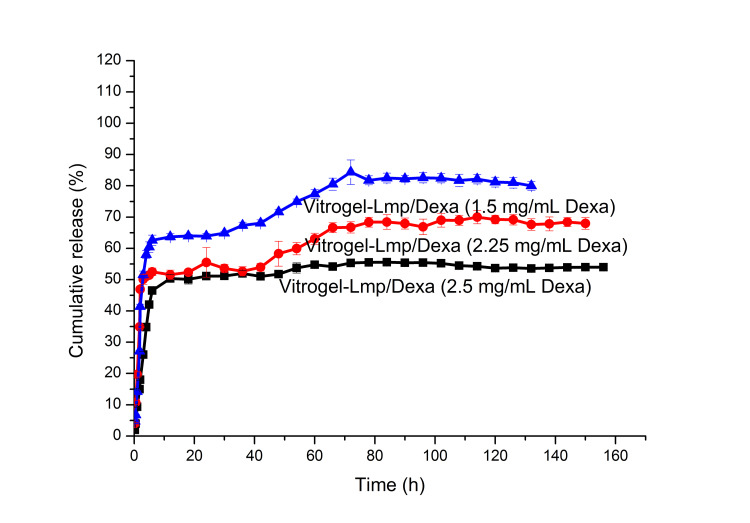
Release profiles of Dexa from Vitrogel-Lmp/Dexa hydrogel samples with different concentrations of Dexa.

Based on the Dexa concentrations determined from the samples collected from the release medium at different time intervals, the cumulative amounts of Dexa released were calculated, and taking into account the Dexa content of the Lmp/Dexa sample subject to release, the cumulative release (%) values were calculated and plotted against time for each of the three different samples (Figure 1). As indicated in the release profile, Dexa was released from Lmp/Dexa-hydrogel continuously for six days in a steady manner. Unlike the in vitro release study from microparticles, in this case, the hydrogel protected the Lmp/Dexa microparticles from degradation. Due to the process of swelling in water, the porous network of the hydrogel favors the transport of drug molecules in the release medium. As a result, the hydrogel network does not behave as a barrier, which prevents the release of Dexa from the embedded microparticles. Although ethanol molecules have the opposite effect of dehydrating the hydrogel network, by competing for the water molecules with which they form stable hydrogen bonds, the very low ethanol content in the release medium only slightly counterbalanced the hydration process, swelling, and, thus, enlarging of the network pores. Therefore, the presence of ethanol can lead to a slight prolongation of the release process. Dexa time release profiles for Vitrogel-Lmp/Dexa hydrogel samples with different concentrations of Dexa are shown in Figure 1. As can be seen from the figure, with a lower concentration of Dexa, a higher percentage of release is obtained in the first 12 hours. A possible explanation for this observation could be that the higher the density of Dexa molecules, the lower the rate of diffusion through the hydrogel network. However, the differences were not significant, in terms of the correlation between Dexa concentration and release rate. The release profiles were similar, with progressive release in the first 12 hours, followed by constant release over a period of up to six days. In the case of the sample with the highest concentration of Dexa, the release profile was more uniform due to there being smaller areas where the degree of loading with Dexa is lower, thus favoring the local increase in the diffusion rate of small drug molecules. The results of the study certainly indicate the prolonged release of Dexa from Vitrogel-Lmp/Dexa (i.e., for six days), which indicates the potential of the system studied in the in vitro and in vivo studies for the development of a suitable and precisely controlled delivery system for inner ear therapy.

In vitro study on the HEI-OC1 cell line

Fluorescence Microscopy

**Figure 2:**
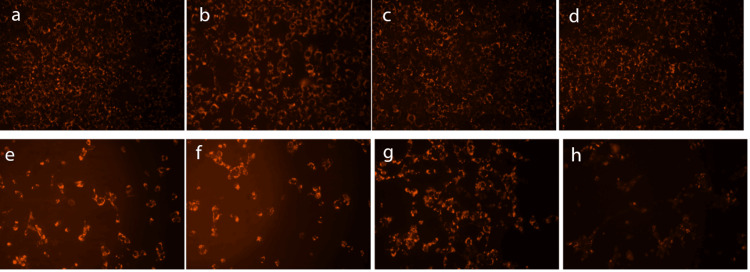
Fluorescence microscopy images of viable HEI-OC1 cells stained with MitoRed (560 nm filter; magnification 20x). a, Control HEI-OC1 cells; b, Dexa; c, Vitrogel-Lmp/Dexa; d, Lmp/Dexa; e, cisplatin; f, Dexa + cisplatin; g, Vitrogel-Lmp/Dexa 15 + cisplatin ; h, Lmp/Dexa + cisplatin.

Viable HEI-OC1 cells showed bright red granulations in the cytoplasm after MitoRed staining. Vitrogel-Lmp/Dexa, Lmp, and Dexa showed no toxicity toward HEI-OC1 cells, as evidenced by fluorescence microscopy (Fig. 2b-2d), while cisplatin treatment reduced the number of viable cells stained with MitoRed (Fig. 2e). HEI-OC1 cells cultured with cisplatin and Dexa 15 µg or Lmp/Dexa showed similar reductions in viability to the cells exposed to cisplatin only (Fig. 2f, 2h). The only treatment that reduced the cytotoxic effect of cisplatin was Vitrogel-Lmp/Dexa 15 (Fig. 2g).

Alamar Blue Test

**Figure 3:**
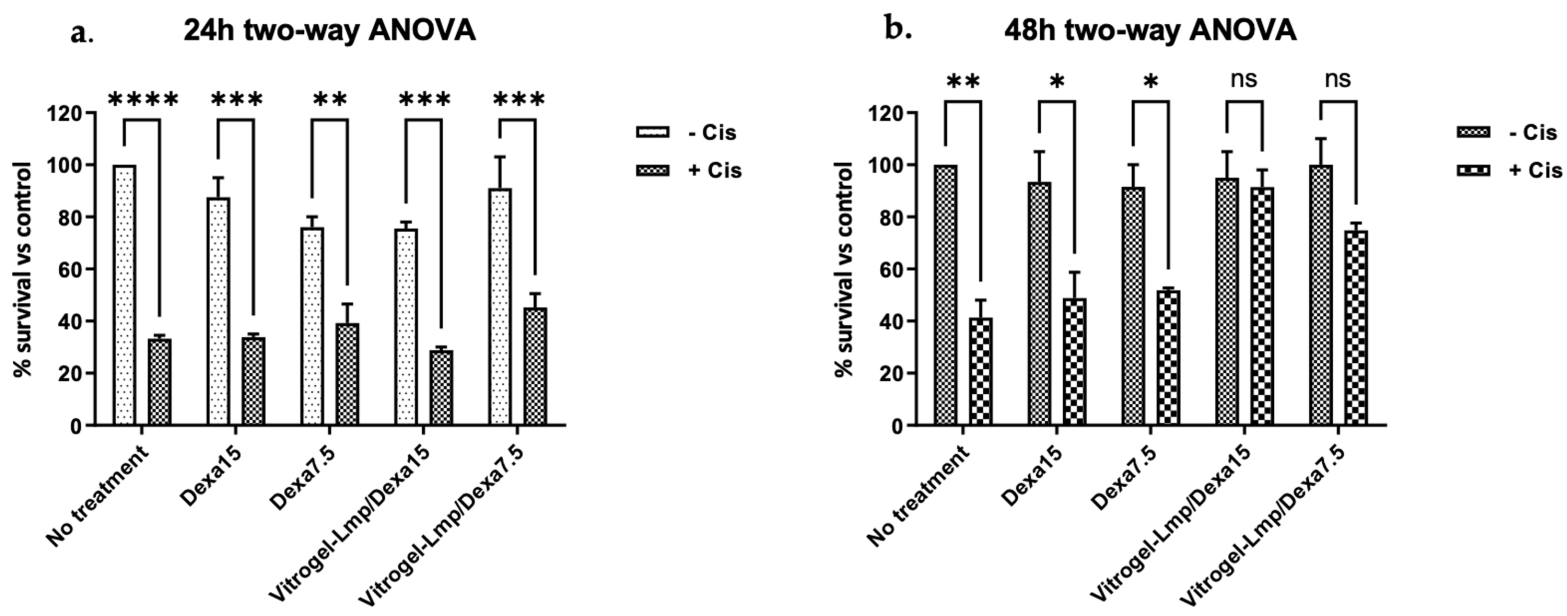
Two-way ANOVA followed by Bonferroni's multiple comparison statistical test of the viability of HEI-OC1 cells after cisplatin treatment; a, 24 h after treatment; b, 48h after treatment. Vitrogel-Lmp/Dexa offered significant protection against cisplatin toxicity after 48 h incubation, with more important results for the Vitrogel-Lmp/Dexa containing 15 µg of Dexa (Vitrogel-Lmp/Dexa15 p>0.9999; Vitrogel-Lmp/Dexa7.5 p=0.2226). The calculated Interaction Value at 24 hours was F(4,20) = 6.089 with p=0.0023 and F(4,10)=3.649 with p=0.0441 at 48 hours.

Treatment with 100 µM cisplatin reduced the percentage of viable cells to 33.25% versus control cells at 24 hours (Fig. 3a) and 41.4% at 48 hours (Fig. 3b). After 24 hours, the cells seeded on Vitrogel-Lmp/Dexa 15 or Vitrogel-Lmp/Dexa 7.5 or treated with free Dexa at two concentrations did not survive significantly better than the cells treated only with cisplatin (two-way ANOVA, Bonferroni’s multiple comparisons test; Fig. 3a). The calculated interaction value at 24 hours was F(4,20) = 6.089 with p = 0.0023.

After 48 hours of incubation, the viability of cells seeded on Vitrogel-Lmp/Dexa and treated with cisplatin was significantly higher than that of cells treated with cisplatin without Vitrogel for both concentrations of Dexa (no statistical difference found between the cells treated with cisplatin and Vitrogel-Lmp/Dexa 15 or Vitrogel-Lmp/Dexa 7,5 and the cells without cisplatin administration; two-way ANOVA, Bonferroni’s multiple-comparison test; Fig. 3b). The p-value calculated by two-way ANOVA followed by Bonferroni's multiple-comparison test for the difference in the viability of the cells seeded on Vitrogel-Lmp/Dexa15 was p > 0.9999 and for the cells seeded on Vitrogel-Lmp/Dexa 7.5 was p = 0.2226. The calculated interaction value at 48 hours was F(4,10) = 3.649 with p = 0.0441.

In vivo study audiological results

Prior to the administration of cisplatin, when included in the study, all of the rats had their hearing thresholds for all of the tested frequencies recorded. 

During the one-week waiting period after cisplatin administration, two rats in the first group (Lmp/Dexa) and one in the second group (Vitrogel-Lmp/Dexa) died. Their deaths were attributed to the toxic effects of cisplatin. Therefore, the final number of rats that had their ABR thresholds determined after one week was five in the first group and six in the second. After testing the surviving rats on the seventh day after cisplatin and treatment administration, statistically significant (p < 0.05) results were obtained.

**Figure 4:**
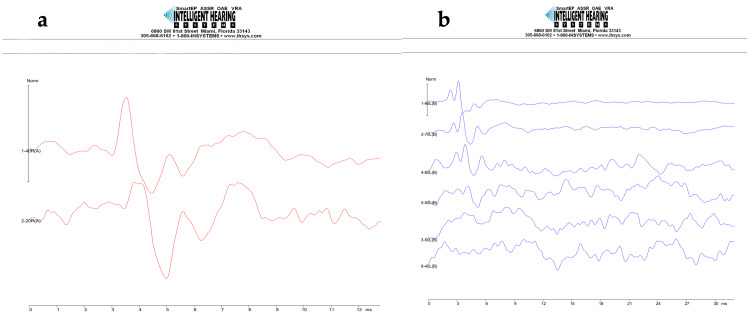
ABR thresholds measured using SmartEP from Intelligent Hearing Systems. Normal thresholds (a) before cisplatin administration and acquired threshold shift (b) after cisplatin administration.

In Figure 4, the results of two ABR tests can be observed: one before and one after administration of cisplatin.

In the Lmp/Dexa group, the tested ear (right ear) of the rats showed a significantly lower hearing threshold shift than the left ear in the 32 kHz frequency (p = 0.0057). For all of the other tested frequencies (8 kHz, 16 kHz, 24kHz, and click stimulus), a difference was observed in absolute values (with lower hearing threshold shift in the right ear), but without statistical significance (p > 0.05). The calculated interaction value in the Lmp/Dexa group was F(4,40) = 1.213 with p = 0.3205.

In the Vitrogel-Lmp/Dexa group, a statistically significant (p < 0.05) acquired ABR threshold shift after seven days was observed in the right ear (tested ear) compared with in the left ear in the 32 kHz (p < 0.0001), 24 kHz (p = 0.0302), 16 kHz (p < 0.0001), and 8 kHz (p = 0.0004) frequencies. In the click stimulus test, there was a difference in absolute values, when comparing the right and left ears, but with no statistical significance (p > 0.05). The calculated interaction value in the Vitrogel-Lmp/Dexa group was F(4,50) = 2.673 with p = 0.0425.

**Figure 5:**
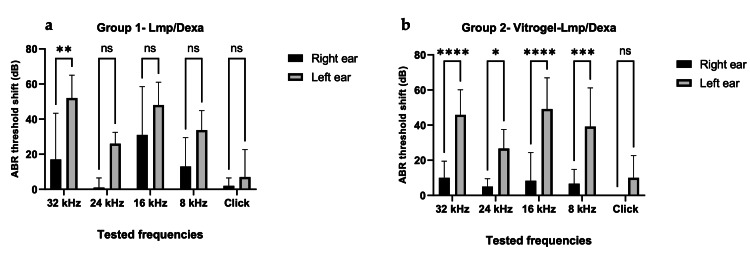
Mean ABR threshold shift (dB) in the Lmp/Dexa (a) and Vitrogel-Lmp/Dexa (b) groups after cisplatin and treatment administration (right ear: tested ear, left ear: control ear) after seven days. Two-way ANOVA followed by Bonferroni's multiple-comparison statistical test shows significant inner ear protection at 32 kHz for Lmp/Dexa (p = 0.0057) (a) and 32 kHz (p < 0.0001), 24 kHz (p = 0.0302), 16 kHz (p < 0.0001), 8 kHz (p = 0.0004) for Vitrogel-Lmp/Dexa (b). The calculated interaction value in the Lmp/Dexa group was F(4,40) = 1.213 with p = 0.3205 (a) and F(4,50) = 2.673 with p = 0.0425 for the Vitrogel-Lmp/Dexa group (b).

In the following figure (Fig. 5), the mean ABR threshold shift for each tested frequency of both groups can be observed. 

In vivo study histological examination results

**Figure 6:**
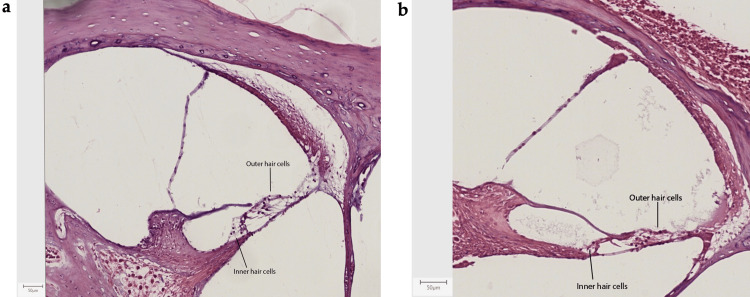
H&E staining results, showing loss of integrity at the level of the epithelial structures of the inner ear (lower number of outer and inner ear hair cells) in the case of treatment with dexamethasone (b) (10X). Normal epithelial structures in the Vitrogel-Lmp/Dexa group (a) (10X).

**Figure 7:**
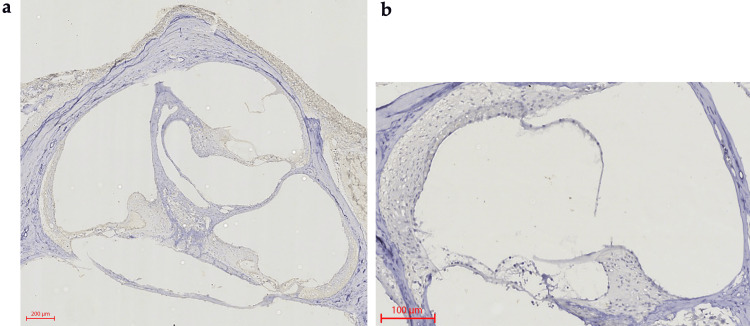
Immunohistochemical staining for procaspase-3: increased cytoplasmic expression of pro-caspase-3 (brown) in the ear treated with free dexamethasone (a) (2,5X) compared with Vitrogel-Lmp/Dexa (b) (10X).

After analyzing the slides using H&E staining, a loss of integrity at the level of the epithelial structures of the inner ear due to the ototoxic effect of cisplatin could be observed in the ears treated with Dexa (control ears). In comparison, the ears treated with Lmp/Dexa and Vitrogel-Lmp/Dexa (both treated ear groups) showed better integrity of the structures, with comparable results in both microcarrier groups (Fig. 6). After immunohistochemical staining for procaspase-3, an increased cytoplasmatic expression of procaspase-3 was observed in the dexamethasone-treated ears (control ears) compared with those treated using the biopolymer microcarrier loaded with dexamethasone (Lmp/Dexa- and Vitrogel-Lmp/Dexa-treated ears, respectively). The increased procaspase-3 activity is highlighted by the brown color in the figure. Increased procaspase-3 staining can be seen diffusely in all of the epithelial structures of the inner ear, except for the otic capsule. Tissues that do not present increased immunohistochemical staining for procaspase-3 activity appear blue. These results demonstrate increased cellular damage and apoptosis in the dexamethasone-treated ears of the rats. Bone was used as a negative control in the immunohistochemical staining analysis (Fig. 7). 

## Discussion

As acquired sensorineural hearing loss and the ototoxic effects of various drugs represent an important problem in the medical world, novel treatment options are continuously being researched. The most significant challenge in treating inner ear disease is represented by the successful delivery of therapeutic agents to the inner ear, particularly without compromising the integrity of its structures. Due to the risks and limitations of systemic drug delivery, researchers have been investigating safe local delivery methods that can deliver drugs without any of the side effects associated with systemic administration. 

In an effort to enhance drug delivery into the inner ear, our research team has previously engineered an innovative microcarrier that can allow for prolonged delivery of dexamethasone at this level [18]. 

In this study, we described an evolution of the previously presented microcarrier. The same microcarrier, when included into a bioactive hydrogel, can provide prolonged delivery of Dexa and prevent the natural clearing of middle ear contents through the Eustachian tube. We demonstrated the effectiveness of the newly described Vitrogel drug delivery system on the HEI-OC1 cell line through fluorescence microscopy with MitoRed stain and an Alamar Blue viability test. These tests revealed that culturing HEI-OC1 cells on Vitrogel-Lmp/Dexa had an important protective effect against cisplatin toxicity, which was significantly stronger than the dexamethasone solution, especially after 48 hours of incubation. These results were in accordance with the in vitro release test, which showed an increased release of Dexa under the Vitrogel-Lmp/Dexa system at this interval compared with that at 24 hours. The superior results obtained with Vitrogel-Lmp/Dexa compared to Lmp/Dexa in the MitoRed Fluorescence microscopy analysis could be explained by the lower cumulative release of dexamethasone from Vitrogel-Lmp/Dexa compared to Lmp/Dexa for the same Dexa concentration, which aligns with the toxicity studies of Lmp/Dexa on HEI-OC1 cells published by our team when testing the toxicity of Lmp/Dexa at different concentrations on HEI-OC1 cells; in particular, it was shown that lower Lmp/Dexa concentrations provide no significant reduction in cell viability when compared to control [18]. In the rat in vivo study, we demonstrated that both the Dexa-loaded microparticles (LMP/Dexa) and the loaded microparticles embedded in Vitrogel (Vitrogel-Lmp/Dexa) offer better protection against cisplatin-induced hearing loss when injected into the middle ear than free dexamethasone solution. Vitrogel-Lmp/Dexa offered significantly better protection against the ototoxic effects of the chemotherapeutic agent for almost all frequencies tested (32 kHz, 24 kHz, 16 kHz, and 8 kHz), while Lmp/Dexa offered significantly better results than dexamethasone solution for the 32 kHz frequency, without any statistically significant protection in the other frequencies tested. The newly described Vitrogel-Lmp/Dexa most probably obtained better results than the microcarrier without Vitrogel due to its advantage of preventing clearance through the Eustachian tube from the middle ear, as mediated by the physical retention of the microcarriers in the middle ear; in turn, this allows for the continuous release of dexamethasone for up to six days.

The results obtained in the HEI-OC1 cell line in this study were backed up by the results obtained in the animal model, which jointly prove that the Vitrogel-Lmp/Dexa is a better solution for protecting against cisplatin-induced hearing loss than free dexamethasone injection when administered locally. 

Intratympanic dexamethasone is known to offer protection against cisplatin ototoxicity. This effect has been shown in an experimental in vivo study in guinea pigs, where dexamethasone injection administered one hour before cisplatin showed a protective effect against cisplatin-induced toxicity at a dose of 8 mg/kg [23]. Our results indicated a better otoprotective effect offered by the prolonged release of the Lmp/Dexa and Vitrogel-Lmp/Dexa than the single-dose injection treatment option described by other authors, with the limitation that the study mentioned above was conducted on guinea pigs, and the described in vivo study was conducted on rats [23,24].

Our results are in accordance with other studies regarding drug release systems in the intracochlear setting. Microparticles loaded with corticosteroids have been tested in combination with a cochlear implant. Fluticasone propionate intracochlear implants with extended release were shown to protect against cisplatin-induced ototoxicity in a guinea pig experimental model, as reported by Pierstorff et al. [25]. Another group studied the possibility of residual hearing preservation after cochlear implant surgery using gold nanoparticles loaded with dexamethasone [26]. The authors observed better preservation of residual hearing after a cochlear implant model in the 8 kHz frequency range when applying the nanoparticles in the round window. No statistically significant differences were found in the other frequency ranges. Lmp/Dexa and Vitrogel-Lmp/Dexa could be used as a treatment option for preserving residual hearing after a cochlear implant, either by direct application or by incorporating the microparticles in drug-eluting electrodes. Further studies are needed to test our drug delivery systems in the context of cochlear implant surgery.

The HEI-OC1 cell line represents a commonly used experimental model for drug ototoxicity research [27]. In a study published by Cervantes et al. in 2019, solid lipid nanoparticles loaded with corticosteroids (dexamethasone and hydrocortisone) provided protection against cisplatin toxicity on HEI-OC1 cells. The authors found increased effectiveness in protecting against cisplatin ototoxicity for the nanoparticles loaded with corticosteroids when compared to simple administration of the drugs [28]. Our results align with the results presented in this in vitro study and, moreover, our in vitro study results are strengthened by the results from the in vivo study in Wistar rats. 

In an in vivo experimental study on rats, dexamethasone and methylprednisolone intratympanic injection were tested for their protective effects against cisplatin intraperitoneal injection (15 mg/kg). The median hearing threshold shift in the control group after cisplatin administration and intratympanic NaCl solution on the tested frequencies was 47 dB; meanwhile, in the intratympanic dexamethasone group, the median hearing threshold shift was 30 dB [24]. In our study, the same intraperitoneal cisplatin dose (15 mg/kgc) led to a mean hearing threshold shift of 12.8 dB in the Lmp/Dexa group, compared to a mean of 6 dB of hearing threshold shift in the Vitrogel-Lmp/Dexa group. In the ears injected with dexamethasone (control ears), we obtained average hearing threshold shifts of 33.4 dB in the Lmp/Dexa group and 34.16 dB in the Vitrogel-Lmp/Dexa group. The median hearing threshold shift represents the middle value of the ABR threshold results, while the mean hearing threshold shift represents the average hearing threshold shift in all of the tested frequencies. The results obtained in our in vivo study were comparable for the control ears (free dexamethasone injection) to other studies published in the literature for the same cisplatin dosage with the same administration route, while the results obtained with the newly described Lmp/Dexa and Vitrogel-Lmp/Dexa were superior, offering better protection than a simple dexamethasone injection. 

While clinical trials have also been performed in humans receiving cisplatin treatment, the results of these studies have been variable. Marshak et al. published the results of a randomized controlled study where intratympanic dexamethasone was administered to patients before cisplatin treatment. Each patient had a studied ear and a control ear. Their conclusion was that intratympanic dexamethasone significantly protected against hearing loss at 6000 Hz and significantly decreased outer hair cell dysfunction in the Distortion Product Otoacoustic Emissions test [29]. A more recent randomized controlled phase IIIB clinical trial tested whether intratympanic dexamethasone administered through a passive diffusion device (Microwick) can protect against cisplatin-induced hearing loss. Intratympanic dexamethasone was administered from the start of the cisplatin treatment to three weeks after the last cycle. In the audiometric analysis, statistically significant differences could be observed at frequencies of 500 (4.9 dB), 1000 (5.5 dB), and 6000 Hz (16 dB), but were not considered clinically significant under the current hearing loss criteria [30]. In the clinical trials, some hearing protection was observed at different tested frequencies, but the results were not as good as the experimental results obtained with the described drug-delivery system. More clinical trials researching intratympanic corticoid therapy with different drug delivery devices and drug dosages are needed in order to find a solution for drug-induced hearing loss in patients undergoing platinum-based chemotherapy treatment. Treatment with Vitrogel-Lmp/Dexa may provide better results than free intratympanic dexamethasone injection used in the aforementioned clinical trials.

Using Lmp/Dexa and especially Vitrogel-Lmp/Dexa in clinical settings can be expected to provide a better alternative to the free dexamethasone solution. The constant release of dexamethasone from the formulation for 6 days, with a higher initial release in the first 12 hours, and the gellification process that does not permit the clearing of the microparticles through the Eustachian tube are important advantages of the proposed drug delivery system. 

Study limitations

One of the main limitations of this study when comparing the effectiveness of Vitrogel-Lmp/Dexa to the Lmp/Dexa formulation is represented by the difference in the number of animals that had their hearing tested through ABR at seven days after cisplatin administration, due to the lower number of animals in the Lmp/Dexa group (five tested animals versus six animals in the Vitrogel-Lmp/Dexa group). This is a consequence of the higher mortality in the Lmp/Dexa group after cisplatin administration (two deaths compared to one in the Vitrogel-Lmp/Dexa group). There is a possibility that if more animals were present in the final tested group, statistical significance could’ve been obtained on more of the tested frequencies in the Lmp/Dexa group. 

Another limitation of the study is that cisplatin treatment has long-term effects, even after the duration of treatment provided by the described microparticles. Long-term assessment using this model would provide a more complete image of cisplatin ototoxicity in our experimental model.

Testing Lmp/Dexa and Vitrogel-Lmp/Dexa in another animal study where assessment of the long-term effects of cisplatin is undertaken could provide important information on the long-term efficacy of the described systems. Clinical trials including the described drug delivery system represent the end goal of our research in order to provide a safe option for preventing cisplatin-induced hearing loss in patients undergoing cisplatin chemotherapy.

## Conclusions

The newly described microcarrier of dexamethasone (Lmp/Dexa), especially the microcarrier combined with a thermosensitive hydrogel (Vitrogel-Lmp/Dexa), offered better protection against cisplatin-induced hearing loss than free dexamethasone, both in vitro (HEI-OC1 cells) and in vivo (Wistar rats). The microcarriers could provide an important treatment option for oncological patients undergoing cisplatin treatment, and they could also be useful in the treatment of other inner ear pathologies where an efficient, prolonged, and local anti-inflammatory treatment is needed.

## References

[1] (2021). Hearing loss prevalence and years lived with disability, 1990-2019: findings from the Global Burden of Disease Study 2019. Lancet.

[2] Livingston G, Huntley J, Sommerlad A (2020). Dementia prevention, intervention, and care: 2020 report of the Lancet Commission. Lancet.

[3] Dindelegan MG, Blebea C, Perde-Schrepler M, Buzoianu AD, Maniu AA (2022). Recent advances and future research directions for hearing loss treatment based on nanoparticles. J Nanomater.

[4] Nieman CL, Oh ES (2020). Hearing loss. Ann Intern Med.

[5] Schellack N, Lecturer S, Naude A, Pathology M, Lecturer S (2013). An overview of pharmacotherapy-induced ototoxicity. S Afr Fam Pract.

[6] Steyger PS (2011). Mechanisms involved in ototoxicity. Semin Hear.

[7] Tang Q, Wang X, Jin H (2021). Cisplatin-induced ototoxicity: Updates on molecular mechanisms and otoprotective strategies. Eur J Pharm Biopharm.

[8] Dillard LK, Lopez-Perez L, Martinez RX, Fullerton AM, Chadha S, McMahon CM (2022). Global burden of ototoxic hearing loss associated with platinum-based cancer treatment: A systematic review and meta-analysis. Cancer Epidemiol.

[9] Callejo A, Sedó-Cabezón L, Juan ID, Llorens J (2015). Cisplatin-induced ototoxicity: effects, mechanisms and protection strategies. Toxics.

[10] Arora R, Thakur JS, Azad RK, Mohindroo NK, Sharma DR, Seam RK (2009). Cisplatin-based chemotherapy: add high-frequency audiometry in the regimen. Indian J Cancer.

[11] Tan WJ, Vlajkovic SM (2023). Molecular characteristics of cisplatin-induced ototoxicity and therapeutic interventions. Int J Mol Sci.

[12] Yu D, Gu J, Chen Y, Kang W, Wang X, Wu H (2020). Current strategies to combat cisplatin-induced ototoxicity. Front Pharmacol.

[13] Hill GW, Morest DK, Parham K (2008). Cisplatin-induced ototoxicity: effect of intratympanic dexamethasone injections. Otol Neurotol.

[14] Salt AN, Plontke SK (2005). Local inner-ear drug delivery and pharmacokinetics. Drug Discov Today.

[15] Williams DM (2018). Clinical pharmacology of corticosteroids. Respir Care.

[16] Dormer NH, Nelson-Brantley J, Staecker H, Berkland CJ (2019). Evaluation of a transtympanic delivery system in Mus musculus for extended release steroids. Eur J Pharm Sci.

[17] Salt AN, Hartsock J, Plontke S, Lebel C, Piu F (2011). Distribution of dexamethasone and preservation of inner ear function following intratympanic delivery of a gel-based formulation. Audiol Neurootol.

[18] Dindelegan MG, Pașcalău V, Suciu M (2022). Biopolymer lipid hybrid microcarrier for transmembrane inner ear delivery of dexamethasone. Gels.

[19] Paşcalău V, Tertis M, Pall E (2020). Bovine serum albumin gel/polyelectrolyte complex of hyaluronic acid and chitosan based microcarriers for Sorafenib targeted delivery. J Appl Polym Sci.

[20] Perde-Schrepler M, Fischer-Fodor E, Virag P (2020). The expression of copper transporters associated with the ototoxicity induced by platinum-based chemotherapeutic agents. Hear Res.

[21] Curzer HJ, Perry G, Wallace MC, Perry D (2016). The three rs of animal research: what they mean for the institutional animal care and use committee and why. Sci Eng Ethics.

[22] Ruebhausen M, Brozoski T, Bauer C (2012). A comparison of the effects of isoflurane and ketamine anesthesia on auditory brainstem response (ABR) thresholds in rats. Hear Res.

[23] Shafik AG, Elkabarity RH, Thabet MT, Soliman NB, Kalleny NK (2013). Effect of intratympanic dexamethasone administration on cisplatin-induced ototoxicity in adult guinea pigs. Auris Nasus Larynx.

[24] Taş BM, Şimşek G, Azman M, Kılıç R (2021). Efficacy of 2 different intratympanic steroid regimen on prevention of cisplatin ototoxicity: an experimental study. Ear Nose Throat J.

[25] Pierstorff E, Yang WW, Chen YA, Cheung S, Kalinec F, Slattery WH (2019). Prevention of cisplatin-induced hearing loss by extended release fluticasone propionate intracochlear implants. Int J Pediatr Otorhinolaryngol.

[26] Blebea CM, Necula V, Potara M (2022). The effect of pluronic-coated gold nanoparticles in hearing preservation following cochlear implantation-pilot study. Audiol Res.

[27] Kalinec G, Thein P, Park C, Kalinec F (2016). HEI-OC1 cells as a model for investigating drug cytotoxicity. Hear Res.

[28] Cervantes B, Arana L, Murillo-Cuesta S, Bruno M, Alkorta I, Varela-Nieto I (2019). Solid lipid nanoparticles loaded with glucocorticoids protect auditory cells from cisplatin-induced ototoxicity. J Clin Med.

[29] Marshak T, Steiner M, Kaminer M, Levy L, Shupak A (2014). Prevention of cisplatin-induced hearing loss by intratympanic dexamethasone: a randomized controlled study. Otolaryngol Head Neck Surg.

[30] Moreno I, Belinchon A (2022). Evaluating the efficacy of intratympanic dexamethasone in protecting against irreversible hearing loss in patients on cisplatin-based cancer treatment: a randomized controlled phase IIIB clinical trial. Ear Hear.

